# Supplementary material for: Resolving the full spectrum of human genome variation using Linked-Reads
Source: Genome Res. 2019 Apr;29(4):635–45. doi: 10.1101/gr.234443.118 (PMC6442396; doi:10.1101/gr.234443.118)
Supplement: Supplemental Material [file supp_gr.234443.118_Supplemental_longranger-2.2.zip › longranger-2.2.tar.bz2!/lr2.2-public/lib/stan/cmdstan/stan/doc/api/html/agrad_2rev_2ode_2coupled__ode__system_8hpp_source.html]

Stan: src/stan/agrad/rev/ode/coupled\_ode\_system.hpp Source File


|  |  |
| --- | --- |
|  | Stan  2.5.0  probability, sampling & optimization |


- Main Page
- Related Pages
- Namespaces
- Classes
- Files

- File List
- File Members

All Classes Namespaces Files Functions Variables Typedefs Enumerations Enumerator Friends Macros Pages

- src
- stan
- agrad
- rev
- ode

coupled\_ode\_system.hpp

Go to the documentation of this file.

1 #ifndef STAN\_\_AGRAD\_\_REV\_\_ODE\_\_COUPLED\_ODE\_SYSTEM\_HPP

2 #define STAN\_\_AGRAD\_\_REV\_\_ODE\_\_COUPLED\_ODE\_SYSTEM\_HPP

3

4 #include <ostream>

5 #include <vector>

6 #include <stan/agrad/rev/functions/value\_of.hpp>

7 #include <stan/agrad/rev/internal/precomputed\_gradients.hpp>

8 #include <stan/agrad/rev/operators/operator\_plus\_equal.hpp>

9 #include <stan/math/error\_handling/check\_equal.hpp>

10 #include <stan/math/error\_handling/matrix/check\_matching\_sizes.hpp>

11 #include <stan/math/ode/coupled\_ode\_system.hpp>

12 #include <stan/meta/traits.hpp>

13

14 namespace stan {

15

16  namespace math {

17

18  // This code is in this directory because it includes agrad::var

19  // It is in namespace stan::math so that the partial template

20  // specializations are treated as such.

21

22

34  void add\_initial\_values(const std::vector<stan::agrad::var>& y0,

35  std::vector<std::vector<stan::agrad::var> >& y) {

36  for (size\_t n = 0; n < y.size(); n++)

37  for (size\_t m = 0; m < y0.size(); m++)

38  y[n][m] += y0[m];

39  }

40

62  template <typename F>

63  struct coupled\_ode\_system <F, double, stan::agrad::var> {

64

65  const F& f\_;

66  const std::vector<double>& y0\_dbl\_;

67  const std::vector<stan::agrad::var>& theta\_;

68  std::vector<double> theta\_dbl\_;

69  const std::vector<double>& x\_;

70  const std::vector<int>& x\_int\_;

71  const int N\_;

72  const int M\_;

73  const int size\_;

74  std::ostream\* msgs\_;

75

88  coupled\_ode\_system(const F& f,

89  const std::vector<double>& y0,

90  const std::vector<stan::agrad::var>& theta,

91  const std::vector<double>& x,

92  const std::vector<int>& x\_int,

93  std::ostream\* msgs)

94  : f\_(f),

95  y0\_dbl\_(y0),

96  theta\_(theta),

97  theta\_dbl\_(theta.size(), 0.0),

98  x\_(x),

99  x\_int\_(x\_int),

100  N\_(y0.size()),

101  M\_(theta.size()),

102  size\_(N\_ + N\_ \* M\_),

103  msgs\_(msgs) {

104

105  for (int m = 0; m < M\_; m++)

106  theta\_dbl\_[m] = stan::agrad::value\_of(theta[m]);

107  }

108

123  void operator()(const std::vector<double>& y,

124  std::vector<double>& dy\_dt,

125  double t) {

126  using std::vector;

127  using stan::agrad::var;

128

129  vector<double> y\_base(y.begin(), y.begin()+N\_);

130  dy\_dt = f\_(t,y\_base,theta\_dbl\_,x\_,x\_int\_,msgs\_);

131  stan::math::check\_equal("coupled\_ode\_system(%1%)",

132  dy\_dt.size(),N\_,"dy\_dt",

133  static\_cast<double\*>(0));

134

135  vector<double> coupled\_sys(N\_ \* M\_);

136  vector<var> theta\_temp;

137  vector<var> y\_temp;

138  vector<var> dy\_dt\_temp;

139  vector<double> grad;

140  vector<var> vars;

141

142  for (int i = 0; i < N\_; i++) {

143  theta\_temp.clear();

144  y\_temp.clear();

145  dy\_dt\_temp.clear();

146  grad.clear();

147  vars.clear();

148  try {

149  stan::agrad::start\_nested();

150  for (int j = 0; j < N\_; j++) {

151  y\_temp.push\_back(y[j]);

152  vars.push\_back(y\_temp[j]);

153  }

154

155  for (int j = 0; j < M\_; j++) {

156  theta\_temp.push\_back(theta\_dbl\_[j]);

157  vars.push\_back(theta\_temp[j]);

158  }

159  dy\_dt\_temp = f\_(t,y\_temp,theta\_temp,x\_,x\_int\_,msgs\_);

160  dy\_dt\_temp[i].grad(vars, grad);

161

162  for (int j = 0; j < M\_; j++) {

163  // orders derivatives by equation (i.e. if there are 2 eqns

164  // (y1, y2) and 2 parameters (a, b), dy\_dt will be ordered as:

165  // dy1\_dt, dy2\_dt, dy1\_da, dy2\_da, dy1\_db, dy2\_db

166  double temp\_deriv = grad[y\_temp.size() + j];

167  for (int k = 0; k < N\_; k++)

168  temp\_deriv += y[N\_ + N\_ \* j + k] \* grad[k];

169

170  coupled\_sys[i + j \* N\_] = temp\_deriv;

171  }

172  } catch (const std::exception& e) {

173  stan::agrad::recover\_memory\_nested();

174  throw;

175  }

176  stan::agrad::recover\_memory\_nested();

177  }

178

179  dy\_dt.insert(dy\_dt.end(), coupled\_sys.begin(), coupled\_sys.end());

180  }

181

187  int size() const {

188  return size\_;

189  }

190

204  std::vector<double> initial\_state() {

205  std::vector<double> state(size\_, 0.0);

206  for (int n = 0; n < N\_; n++)

207  state[n] = y0\_dbl\_[n];

208  return state;

209  }

210

211

218  std::vector<std::vector<stan::agrad::var> >

219  decouple\_states(const std::vector<std::vector<double> >& y) {

220  using stan::agrad::precomputed\_gradients;

221  std::vector<stan::agrad::var> temp\_vars;

222  std::vector<double> temp\_gradients;

223  std::vector<std::vector<stan::agrad::var> > y\_return(y.size());

224

225  for (size\_t i = 0; i < y.size(); i++) {

226  temp\_vars.clear();

227

228  //iterate over number of equations

229  for (size\_t j = 0; j < N\_; j++) {

230  temp\_gradients.clear();

231

232  //iterate over parameters for each equation

233  for (size\_t k = 0; k < M\_; k++)

234  temp\_gradients.push\_back(y[i][y0\_dbl\_.size()

235  + y0\_dbl\_.size() \* k + j]);

236

237  temp\_vars.push\_back(precomputed\_gradients(y[i][j],

238  theta\_,

239  temp\_gradients));

240  }

241  y\_return[i] = temp\_vars;

242  }

243

244  return y\_return;

245  }

246

247  };

248

249

250

251

252

253

280  template <typename F>

281  struct coupled\_ode\_system <F, stan::agrad::var, double> {

282

283  const F& f\_;

284  const std::vector<stan::agrad::var>& y0\_;

285  std::vector<double> y0\_dbl\_;

286  const std::vector<double>& theta\_dbl\_;

287  const std::vector<double>& x\_;

288  const std::vector<int>& x\_int\_;

289  std::ostream\* msgs\_;

290  const int N\_;

291  const int M\_;

292  const int size\_;

293

307  coupled\_ode\_system(const F& f,

308  const std::vector<stan::agrad::var>& y0,

309  const std::vector<double>& theta,

310  const std::vector<double>& x,

311  const std::vector<int>& x\_int,

312  std::ostream\* msgs)

313  : f\_(f),

314  y0\_(y0),

315  y0\_dbl\_(y0.size(), 0.0),

316  theta\_dbl\_(theta),

317  x\_(x),

318  x\_int\_(x\_int),

319  msgs\_(msgs),

320  N\_(y0.size()),

321  M\_(theta.size()),

322  size\_(N\_ + N\_ \* N\_) {

323

324  for (int n = 0; n < N\_; n++)

325  y0\_dbl\_[n] = stan::agrad::value\_of(y0\_[n]);

326  }

327

341  void operator()(const std::vector<double>& y,

342  std::vector<double>& dy\_dt,

343  double t) {

344  std::vector<double> y\_base(y.begin(), y.begin() + N\_);

345  for (int n = 0; n < N\_; n++)

346  y\_base[n] += y0\_dbl\_[n];

347

348  dy\_dt = f\_(t,y\_base,theta\_dbl\_,x\_,x\_int\_,msgs\_);

349  stan::math::check\_equal("coupled\_ode\_system(%1%)",

350  dy\_dt.size(),N\_,"dy\_dt",

351  static\_cast<double\*>(0));

352

353  std::vector<double> coupled\_sys(N\_ \* N\_);

354

355  std::vector<stan::agrad::var> y\_temp;

356  std::vector<stan::agrad::var> dy\_dt\_temp;

357  std::vector<double> grad;

358  std::vector<stan::agrad::var> vars;

359

360  for (int i = 0; i < N\_; i++) {

361  y\_temp.clear();

362  dy\_dt\_temp.clear();

363  grad.clear();

364  vars.clear();

365  try {

366  stan::agrad::start\_nested();

367  for (int j = 0; j < N\_; j++) {

368  y\_temp.push\_back(y[j] + y0\_dbl\_[j]);

369  vars.push\_back(y\_temp[j]);

370  }

371

372  dy\_dt\_temp = f\_(t,y\_temp,theta\_dbl\_,x\_,x\_int\_,msgs\_);

373  dy\_dt\_temp[i].grad(vars, grad);

374

375  for (int j = 0; j < N\_; j++) {

376  // orders derivatives by equation (i.e. if there are 2 eqns

377  // (y1, y2) and 2 parameters (a, b), dy\_dt will be ordered as:

378  // dy1\_dt, dy2\_dt, dy1\_da, dy2\_da, dy1\_db, dy2\_db

379  double temp\_deriv = grad[j];

380  for (int k = 0; k < N\_; k++)

381  temp\_deriv += y[N\_ + N\_ \* j + k] \* grad[k];

382

383  coupled\_sys[i+j\*N\_] = temp\_deriv;

384  }

385  } catch (const std::exception& e) {

386  stan::agrad::recover\_memory\_nested();

387  throw;

388  }

389  stan::agrad::recover\_memory\_nested();

390  }

391

392  dy\_dt.insert(dy\_dt.end(), coupled\_sys.begin(), coupled\_sys.end());

393  }

394

400  int size() const {

401  return size\_;

402  }

403

418  std::vector<double> initial\_state() {

419  return std::vector<double>(size\_, 0.0);

420  }

421

429  std::vector<std::vector<stan::agrad::var> >

430  decouple\_states(const std::vector<std::vector<double> >& y) {

431  using stan::agrad::precomputed\_gradients;

432  using stan::agrad::var;

433  using std::vector;

434

435  vector<var> temp\_vars;

436  vector<double> temp\_gradients;

437  vector<vector<var> > y\_return(y.size());

438

439  for (size\_t i = 0; i < y.size(); i++) {

440  temp\_vars.clear();

441

442  // iterate over number of equations

443  for (size\_t j = 0; j < N\_; j++) {

444  temp\_gradients.clear();

445

446  // iterate over parameters for each equation

447  for (size\_t k = 0; k < N\_; k++)

448  temp\_gradients.push\_back(y[i][y0\_.size() + y0\_.size() \* k + j]);

449

450  temp\_vars.push\_back(precomputed\_gradients(y[i][j],

451  y0\_, temp\_gradients));

452  }

453

454  y\_return[i] = temp\_vars;

455  }

456

457  add\_initial\_values(y0\_, y\_return);

458

459  return y\_return;

460  }

461

462  };

463

464

465

466

467

468

469

505  template <typename F>

506  struct coupled\_ode\_system <F, stan::agrad::var, stan::agrad::var> {

507  const F& f\_;

508  const std::vector<stan::agrad::var>& y0\_;

509  std::vector<double> y0\_dbl\_;

510  const std::vector<stan::agrad::var>& theta\_;

511  std::vector<double> theta\_dbl\_;

512  const std::vector<double>& x\_;

513  const std::vector<int>& x\_int\_;

514  const int N\_;

515  const int M\_;

516  const int size\_;

517  std::ostream\* msgs\_;

518

532  coupled\_ode\_system(const F& f,

533  const std::vector<stan::agrad::var>& y0,

534  const std::vector<stan::agrad::var>& theta,

535  const std::vector<double>& x,

536  const std::vector<int>& x\_int,

537  std::ostream\* msgs)

538  : f\_(f),

539  y0\_(y0),

540  y0\_dbl\_(y0.size(), 0.0),

541  theta\_(theta),

542  theta\_dbl\_(theta.size(), 0.0),

543  x\_(x),

544  x\_int\_(x\_int),

545  N\_(y0.size()),

546  M\_(theta.size()),

547  size\_(N\_ + N\_ \* (N\_ + M\_)),

548  msgs\_(msgs) {

549

550  for (int n = 0; n < N\_; n++)

551  y0\_dbl\_[n] = stan::agrad::value\_of(y0[n]);

552

553  for (int m = 0; m < M\_; m++)

554  theta\_dbl\_[m] = stan::agrad::value\_of(theta[m]);

555  }

556

570  void operator()(const std::vector<double>& y,

571  std::vector<double>& dy\_dt,

572  double t) {

573  using std::vector;

574  using stan::agrad::var;

575

576  vector<double> y\_base(y.begin(), y.begin()+N\_);

577  for (int n = 0; n < N\_; n++)

578  y\_base[n] += y0\_dbl\_[n];

579

580  dy\_dt = f\_(t,y\_base,theta\_dbl\_,x\_,x\_int\_,msgs\_);

581  stan::math::check\_equal("coupled\_ode\_system(%1%)",

582  dy\_dt.size(),N\_,"dy\_dt",

583  static\_cast<double\*>(0));

584

585  vector<double> coupled\_sys(N\_ \* (N\_ + M\_));

586  vector<var> theta\_temp;

587  vector<var> y\_temp;

588  vector<var> dy\_dt\_temp;

589  vector<double> grad;

590  vector<var> vars;

591

592  for (int i = 0; i < N\_; i++) {

593  theta\_temp.clear();

594  y\_temp.clear();

595  dy\_dt\_temp.clear();

596  grad.clear();

597  vars.clear();

598  try {

599  stan::agrad::start\_nested();

600

601  for (int j = 0; j < N\_; j++) {

602  y\_temp.push\_back(y[j] + y0\_dbl\_[j]);

603  vars.push\_back(y\_temp[j]);

604  }

605

606  for (int j = 0; j < M\_; j++) {

607  theta\_temp.push\_back(theta\_dbl\_[j]);

608  vars.push\_back(theta\_temp[j]);

609  }

610

611  dy\_dt\_temp = f\_(t,y\_temp,theta\_temp,x\_,x\_int\_,msgs\_);

612  dy\_dt\_temp[i].grad(vars, grad);

613

614  for (int j = 0; j < N\_+M\_; j++) {

615  // orders derivatives by equation (i.e. if there are 2 eqns

616  // (y1, y2) and 2 parameters (a, b), dy\_dt will be ordered as:

617  // dy1\_dt, dy2\_dt, dy1\_da, dy2\_da, dy1\_db, dy2\_db

618  double temp\_deriv = grad[j];

619  for (int k = 0; k < N\_; k++)

620  temp\_deriv += y[N\_ + N\_ \* j + k] \* grad[k];

621

622  coupled\_sys[i + j \* N\_] = temp\_deriv;

623  }

624  } catch (const std::exception& e) {

625  stan::agrad::recover\_memory\_nested();

626  throw;

627  }

628  stan::agrad::recover\_memory\_nested();

629  }

630  dy\_dt.insert(dy\_dt.end(), coupled\_sys.begin(), coupled\_sys.end());

631  }

632

638  int size() const {

639  return size\_;

640  }

641

653  std::vector<double> initial\_state() {

654  return std::vector<double>(size\_, 0.0);

655  }

656

664  std::vector<std::vector<stan::agrad::var> >

665  decouple\_states(const std::vector<std::vector<double> >& y) {

666  using std::vector;

667  using stan::agrad::var;

668  using stan::agrad::precomputed\_gradients;

669

670  vector<var> vars = y0\_;

671  vars.insert(vars.end(), theta\_.begin(), theta\_.end());

672

673  vector<var> temp\_vars;

674  vector<double> temp\_gradients;

675  vector<vector<var> > y\_return(y.size());

676

677  for (size\_t i = 0; i < y.size(); i++) {

678  temp\_vars.clear();

679

680  //iterate over number of equations

681  for (size\_t j = 0; j < N\_; j++) {

682  temp\_gradients.clear();

683

684  //iterate over parameters for each equation

685  for (size\_t k = 0; k < N\_ + M\_; k++)

686  temp\_gradients.push\_back(y[i][N\_ + N\_ \* k + j]);

687

688  temp\_vars.push\_back(precomputed\_gradients(y[i][j],

689  vars, temp\_gradients));

690  }

691  y\_return[i] = temp\_vars;

692  }

693  add\_initial\_values(y0\_, y\_return);

694  return y\_return;

695  }

696

697  };

698

699

700  }

701

702 }

703

704 #endif

stan::math::coupled\_ode\_system< F, stan::agrad::var, stan::agrad::var >::N\_

const int N\_

**Definition:** coupled\_ode\_system.hpp:514

stan::math::check\_equal

bool check\_equal(const char \*function, const T\_y &y, const T\_eq &eq, const char \*name, T\_result \*result)

**Definition:** check\_equal.hpp:57

stan::math::coupled\_ode\_system< F, double, stan::agrad::var >::y0\_dbl\_

const std::vector< double > & y0\_dbl\_

**Definition:** coupled\_ode\_system.hpp:66

N\_

int N\_

**Definition:** mdivide\_left.hpp:20

stan::math::coupled\_ode\_system< F, stan::agrad::var, double >::msgs\_

std::ostream \* msgs\_

**Definition:** coupled\_ode\_system.hpp:289

stan::math::coupled\_ode\_system< F, double, stan::agrad::var >::M\_

const int M\_

**Definition:** coupled\_ode\_system.hpp:72

stan::math::coupled\_ode\_system< F, stan::agrad::var, stan::agrad::var >::theta\_

const std::vector< stan::agrad::var > & theta\_

**Definition:** coupled\_ode\_system.hpp:510

stan::math::coupled\_ode\_system< F, double, stan::agrad::var >::size

int size() const

Returns the size of the coupled system.

**Definition:** coupled\_ode\_system.hpp:187

stan::math::coupled\_ode\_system< F, stan::agrad::var, double >::N\_

const int N\_

**Definition:** coupled\_ode\_system.hpp:290

stan::agrad::start\_nested

static void start\_nested()

Record the current position so that recover\_memory\_nested() can find it.

**Definition:** var\_stack.hpp:96

stan::math::coupled\_ode\_system< F, stan::agrad::var, double >::x\_int\_

const std::vector< int > & x\_int\_

**Definition:** coupled\_ode\_system.hpp:288

stan::agrad::precomputed\_gradients

var precomputed\_gradients(const double value, const std::vector< var > &vars, const std::vector< double > &gradients)

This function is provided for Stan users that want to compute gradients without using Stan's auto-dif...

**Definition:** precomputed\_gradients.hpp:86

stan::math::coupled\_ode\_system< F, stan::agrad::var, double >::y0\_dbl\_

std::vector< double > y0\_dbl\_

**Definition:** coupled\_ode\_system.hpp:285

stan::math::coupled\_ode\_system< F, stan::agrad::var, stan::agrad::var >::size

int size() const

Returns the size of the coupled system.

**Definition:** coupled\_ode\_system.hpp:638

stan::math::coupled\_ode\_system< F, stan::agrad::var, double >::f\_

const F & f\_

**Definition:** coupled\_ode\_system.hpp:283

coupled\_ode\_system.hpp

stan::math::coupled\_ode\_system< F, stan::agrad::var, stan::agrad::var >::f\_

const F & f\_

**Definition:** coupled\_ode\_system.hpp:507

stan::agrad::grad

static void grad(chainable \*vi)

Compute the gradient for all variables starting from the specified root variable implementation.

**Definition:** chainable.hpp:110

stan::math::coupled\_ode\_system< F, stan::agrad::var, stan::agrad::var >::x\_

const std::vector< double > & x\_

**Definition:** coupled\_ode\_system.hpp:512

stan::math::coupled\_ode\_system< F, stan::agrad::var, stan::agrad::var >::decouple\_states

std::vector< std::vector< stan::agrad::var > > decouple\_states(const std::vector< std::vector< double > > &y)

Return the basic ODE solutions given the specified coupled system solutions, including the partials v...

**Definition:** coupled\_ode\_system.hpp:665

check\_matching\_sizes.hpp

stan::math::coupled\_ode\_system< F, double, stan::agrad::var >::size\_

const int size\_

**Definition:** coupled\_ode\_system.hpp:73

stan::agrad::value\_of

T value\_of(const fvar< T > &v)

Return the value of the specified variable.

**Definition:** value\_of.hpp:16

stan::math::coupled\_ode\_system< F, stan::agrad::var, stan::agrad::var >::msgs\_

std::ostream \* msgs\_

**Definition:** coupled\_ode\_system.hpp:517

stan::math::coupled\_ode\_system< F, stan::agrad::var, double >::size

int size() const

Returns the size of the coupled system.

**Definition:** coupled\_ode\_system.hpp:400

stan::math::coupled\_ode\_system< F, double, stan::agrad::var >::x\_int\_

const std::vector< int > & x\_int\_

**Definition:** coupled\_ode\_system.hpp:70

stan::math::coupled\_ode\_system< F, double, stan::agrad::var >::coupled\_ode\_system

coupled\_ode\_system(const F &f, const std::vector< double > &y0, const std::vector< stan::agrad::var > &theta, const std::vector< double > &x, const std::vector< int > &x\_int, std::ostream \*msgs)

Construct a coupled ODE system with the specified base ODE system, base initial state, parameters, data, and a message stream.

**Definition:** coupled\_ode\_system.hpp:88

stan::math::coupled\_ode\_system< F, stan::agrad::var, stan::agrad::var >::y0\_

const std::vector< stan::agrad::var > & y0\_

**Definition:** coupled\_ode\_system.hpp:508

traits.hpp

stan::math::coupled\_ode\_system< F, double, stan::agrad::var >::N\_

const int N\_

**Definition:** coupled\_ode\_system.hpp:71

stan::math::coupled\_ode\_system< F, stan::agrad::var, double >::coupled\_ode\_system

coupled\_ode\_system(const F &f, const std::vector< stan::agrad::var > &y0, const std::vector< double > &theta, const std::vector< double > &x, const std::vector< int > &x\_int, std::ostream \*msgs)

Construct a coupled ODE system for an unknown initial state and known parameters givne the specified ...

**Definition:** coupled\_ode\_system.hpp:307

stan::math::coupled\_ode\_system< F, double, stan::agrad::var >::operator()

void operator()(const std::vector< double > &y, std::vector< double > &dy\_dt, double t)

Assign the derivative vector with the system derivatives at the specified state and time...

**Definition:** coupled\_ode\_system.hpp:123

stan::math::coupled\_ode\_system< F, stan::agrad::var, double >::initial\_state

std::vector< double > initial\_state()

Returns the initial state of the coupled system.

**Definition:** coupled\_ode\_system.hpp:418

stan::math::coupled\_ode\_system< F, stan::agrad::var, stan::agrad::var >::operator()

void operator()(const std::vector< double > &y, std::vector< double > &dy\_dt, double t)

Populates the derivative vector with derivatives of the coupled ODE system state with respect to time...

**Definition:** coupled\_ode\_system.hpp:570

stan::math::coupled\_ode\_system< F, double, stan::agrad::var >::decouple\_states

std::vector< std::vector< stan::agrad::var > > decouple\_states(const std::vector< std::vector< double > > &y)

Returns the base ODE system state corresponding to the specified coupled system state.

**Definition:** coupled\_ode\_system.hpp:219

check\_equal.hpp

stan::math::coupled\_ode\_system< F, stan::agrad::var, stan::agrad::var >::size\_

const int size\_

**Definition:** coupled\_ode\_system.hpp:516

stan::math::coupled\_ode\_system< F, double, stan::agrad::var >::msgs\_

std::ostream \* msgs\_

**Definition:** coupled\_ode\_system.hpp:74

stan::math::add\_initial\_values

void add\_initial\_values(const std::vector< stan::agrad::var > &y0, std::vector< std::vector< stan::agrad::var > > &y)

Increment the state derived from the coupled system in the with the original initial state...

**Definition:** coupled\_ode\_system.hpp:34

precomputed\_gradients.hpp

stan::math::coupled\_ode\_system< F, double, stan::agrad::var >::theta\_

const std::vector< stan::agrad::var > & theta\_

**Definition:** coupled\_ode\_system.hpp:67

stan::math::coupled\_ode\_system< F, double, stan::agrad::var >::theta\_dbl\_

std::vector< double > theta\_dbl\_

**Definition:** coupled\_ode\_system.hpp:68

stan::math::coupled\_ode\_system< F, stan::agrad::var, stan::agrad::var >::M\_

const int M\_

**Definition:** coupled\_ode\_system.hpp:515

stan::math::coupled\_ode\_system< F, stan::agrad::var, double >::size\_

const int size\_

**Definition:** coupled\_ode\_system.hpp:292

stan::math::coupled\_ode\_system< F, stan::agrad::var, double >::decouple\_states

std::vector< std::vector< stan::agrad::var > > decouple\_states(const std::vector< std::vector< double > > &y)

Return the solutions to the basic ODE system, including appropriate autodiff partial derivatives...

**Definition:** coupled\_ode\_system.hpp:430

stan::agrad::var

Independent (input) and dependent (output) variables for gradients.

**Definition:** var.hpp:27

stan::math::e

double e()

Return the base of the natural logarithm.

**Definition:** constants.hpp:86

stan::math::coupled\_ode\_system< F, stan::agrad::var, stan::agrad::var >::y0\_dbl\_

std::vector< double > y0\_dbl\_

**Definition:** coupled\_ode\_system.hpp:509

stan::math::size

int size(const std::vector< T > &x)

**Definition:** size.hpp:11

size\_

size\_t size\_

**Definition:** dot\_self.hpp:18

stan::math::coupled\_ode\_system

Base template class for a coupled ordinary differential equation system, which adds sensitivities to ...

**Definition:** coupled\_ode\_system.hpp:27

stan::math::coupled\_ode\_system< F, stan::agrad::var, double >::theta\_dbl\_

const std::vector< double > & theta\_dbl\_

**Definition:** coupled\_ode\_system.hpp:286

stan::math::coupled\_ode\_system< F, double, stan::agrad::var >::x\_

const std::vector< double > & x\_

**Definition:** coupled\_ode\_system.hpp:69

operator\_plus\_equal.hpp

stan::math::coupled\_ode\_system< F, stan::agrad::var, double >::M\_

const int M\_

**Definition:** coupled\_ode\_system.hpp:291

stan::agrad::recover\_memory\_nested

static void recover\_memory\_nested()

Recover only the memory used for the top nested call.

**Definition:** var\_stack.hpp:72

stan::math::coupled\_ode\_system< F, stan::agrad::var, stan::agrad::var >::initial\_state

std::vector< double > initial\_state()

Returns the initial state of the coupled system.

**Definition:** coupled\_ode\_system.hpp:653

stan::math::coupled\_ode\_system< F, double, stan::agrad::var >::initial\_state

std::vector< double > initial\_state()

Returns the initial state of the coupled system.

**Definition:** coupled\_ode\_system.hpp:204

stan::math::coupled\_ode\_system< F, stan::agrad::var, double >::x\_

const std::vector< double > & x\_

**Definition:** coupled\_ode\_system.hpp:287

stan::math::coupled\_ode\_system< F, stan::agrad::var, stan::agrad::var >::x\_int\_

const std::vector< int > & x\_int\_

**Definition:** coupled\_ode\_system.hpp:513

stan::math::coupled\_ode\_system< F, stan::agrad::var, stan::agrad::var >::coupled\_ode\_system

coupled\_ode\_system(const F &f, const std::vector< stan::agrad::var > &y0, const std::vector< stan::agrad::var > &theta, const std::vector< double > &x, const std::vector< int > &x\_int, std::ostream \*msgs)

Construct a coupled ODE system with unknown initial value and known parameters, given the base ODE sy...

**Definition:** coupled\_ode\_system.hpp:532

stan::math::coupled\_ode\_system< F, stan::agrad::var, stan::agrad::var >::theta\_dbl\_

std::vector< double > theta\_dbl\_

**Definition:** coupled\_ode\_system.hpp:511

value\_of.hpp

stan::math::coupled\_ode\_system< F, stan::agrad::var, double >::operator()

void operator()(const std::vector< double > &y, std::vector< double > &dy\_dt, double t)

Calculates the derivative of the coupled ode system with respect to the state y at time t...

**Definition:** coupled\_ode\_system.hpp:341

M\_

int M\_

**Definition:** mdivide\_left.hpp:19

stan::math::coupled\_ode\_system< F, stan::agrad::var, double >::y0\_

const std::vector< stan::agrad::var > & y0\_

**Definition:** coupled\_ode\_system.hpp:284

stan::math::coupled\_ode\_system< F, double, stan::agrad::var >::f\_

const F & f\_

**Definition:** coupled\_ode\_system.hpp:65


---

[ Stan Home Page ]

*© 2011–2014,
Stan Development Team.*
